# Supplementary material for: Systematic identification of novel regulatory interactions controlling biofilm formation in the bacterium Escherichia coli
Source: Sci Rep. 2017 Dec 1;7:16768. doi: 10.1038/s41598-017-17114-6 (PMC5711951; doi:10.1038/s41598-017-17114-6)
Supplement: Supplementary file 1 — Supplementary Information [file 41598_2017_17114_MOESM1_ESM.pdf]

Systematic identification of novel regulatory interactions controlling biofilm formation in the bacterium *Escherichia coli*

Gerardo Ruiz Amores[1], Aitor de las Heras[2,3], Ananda Sanches-Medeiros[1], Alistair Elfick[2,3] and Rafael Silva-Rocha[1]*

[1]FMRP - University of São Paulo, Ribeirão Preto, SP, Brazil
[2]Institute for Bioengineering, School of Engineering, University of Edinburgh, Edinburgh, UK
[3]SynthSys Research Centre, University of Edinburgh, Edinburgh, UK

Supporting Information

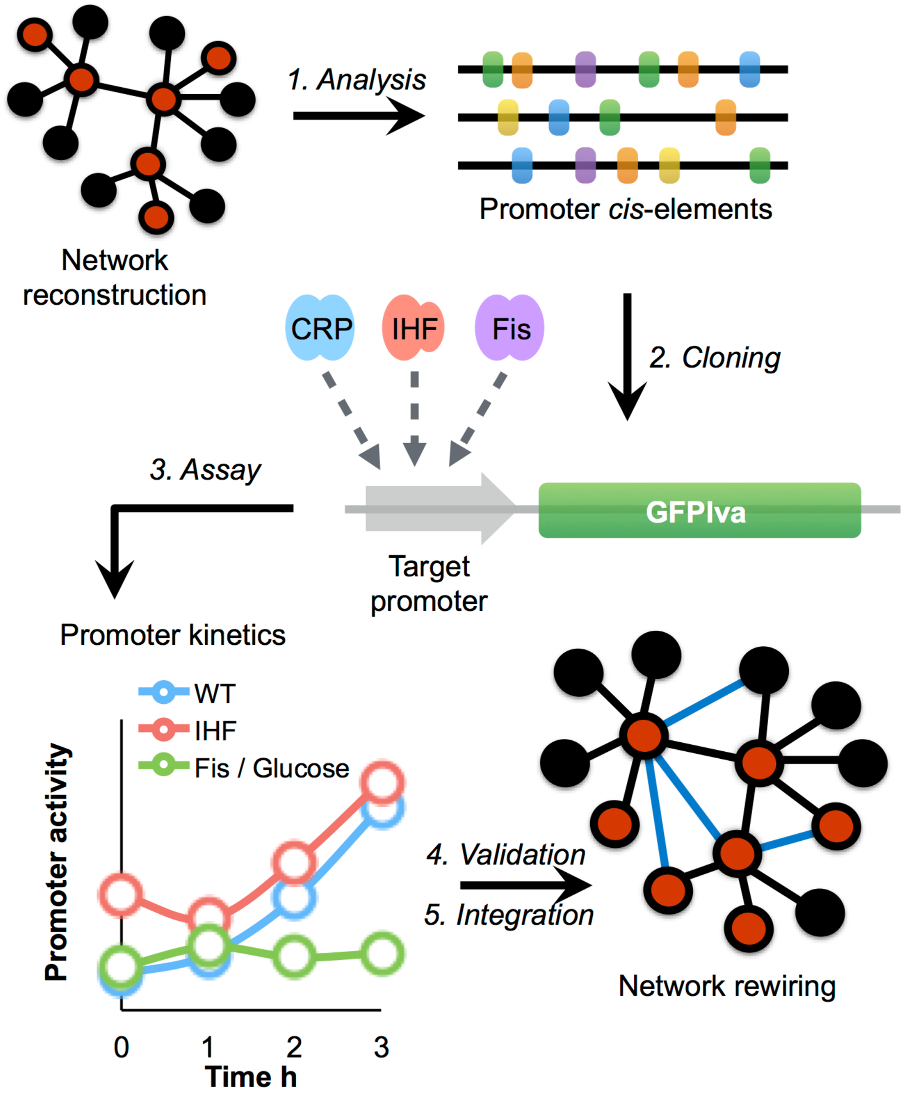

Figure S1. General strategy to define novel regulatory interactions controlling planktonic/biofilm transition. The flagella-biofilm network was constructed and analyzed based on available data from the literature. Subsequently, analysis of the promoter architecture regions of the principal nodes effectors was performed to confirm the interactions reported. Next, cloning of the natural promoter regions in the pMR1-reporter system were developed. Promoter activity was determined for each promoter in different

conditions as established in material and methods. Finally, GFP activity was transformed into connectivity data and loaded into flagella-biofilm network to gain a better understanding of this program.

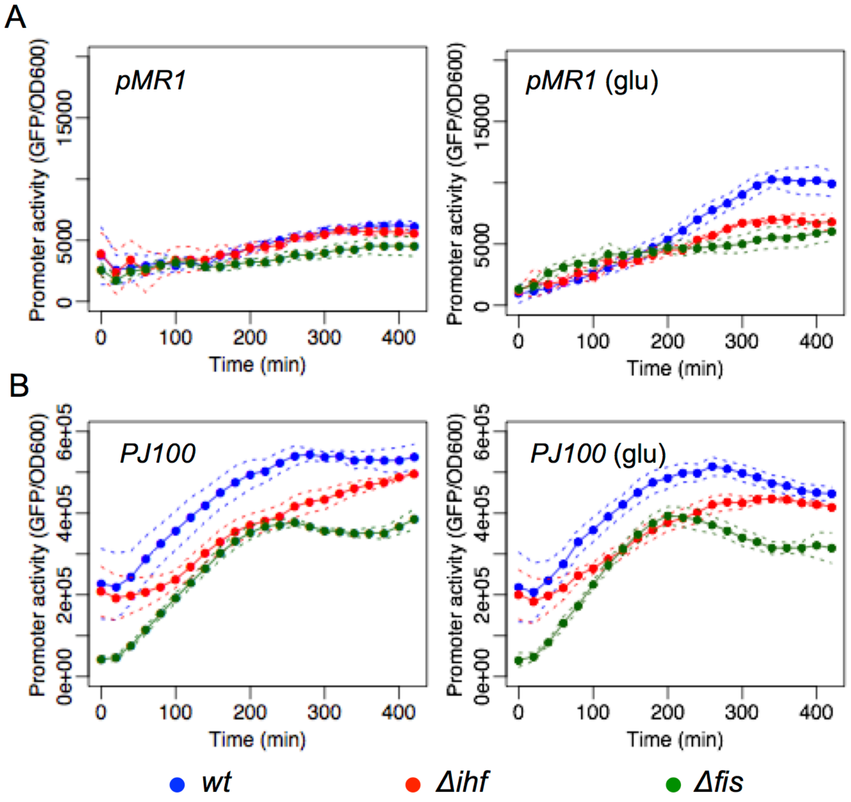

Figure S2. Effect of CRP, IHF and Fis GRs over the controls. Promoter activity assay of (A) pMR1 empty vector and (B) pMR1-*Pj100*, a synthetic constitutive strong promoter. The systems were evaluated in *E. coli* BW25113 *wild-type* (blue line), Δ*ihf* (red line) and Δ*fis* (green line) in 96-well plate as described in methods in the absence (left panel) or presence (right panel) of 0.4% of glucose. GFP fluorescence was measured every 20 minutes over 8 hours growth at 37 ºC in static conditions (normalized by OD600). Solid lines represent the mean of three independent experiments, while dashed lines are the upper and lower limits of standard error (SE) of the mean.

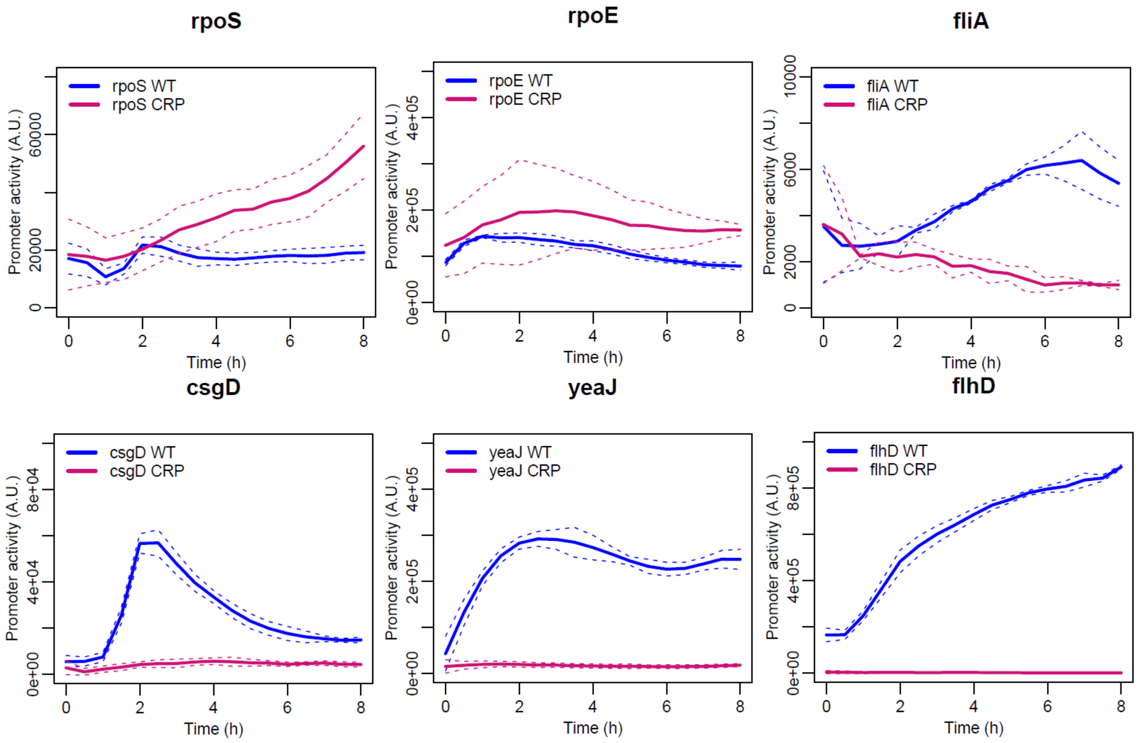

Figure S3. Expression profile of selected promoters in *wild-type* and Δ*crp* mutant strains of *E. coli*. The systems were evaluated in *E. coli* BW25113 *wild-type* (blue line), Δ*crp* (pink line) in 96-well plate as described in methods in the absence of glucose. GFP fluorescence was measured every 20 minutes over 8 hours growth at 37 ºC in static conditions (normalized by OD600). Solid lines represent the mean of three independent experiments, while dashed lines are the upper and lower limits of standard error (SE) of the mean.

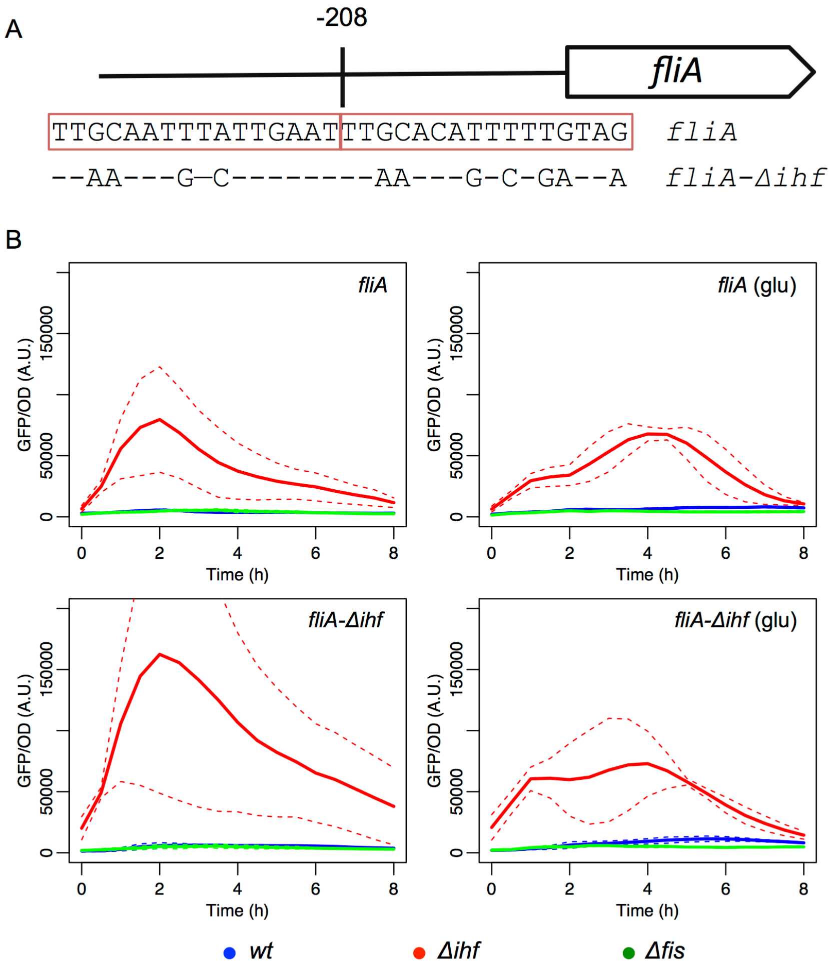

Figure S4. Effect of CRP, IHF and Fis on wild-type and mutant versions of *fliA* promoter. (A) Identification of two putative IHF binding sites at the *fliA* promoter using Virtual Footprint[1]. The inserts represented the original and mutated versions constructed (the putative IHF sites are boxed). (B) Assays of the two promoters were evaluated in *E. coli* BW25113 *wild-type* (blue line), Δ*ihf* (red line) and Δ*fis* (green line) in 96-well plate as described in methods in the absence (left panels) or presence (right panels) of 0.4% of glucose. GFP fluorescence was measured every 20 minutes over 8 hours growth at 37 ºC in static conditions (normalized by OD600). Solid lines represent the mean of three independent experiments, while dashed lines are the upper and lower limits of SE.

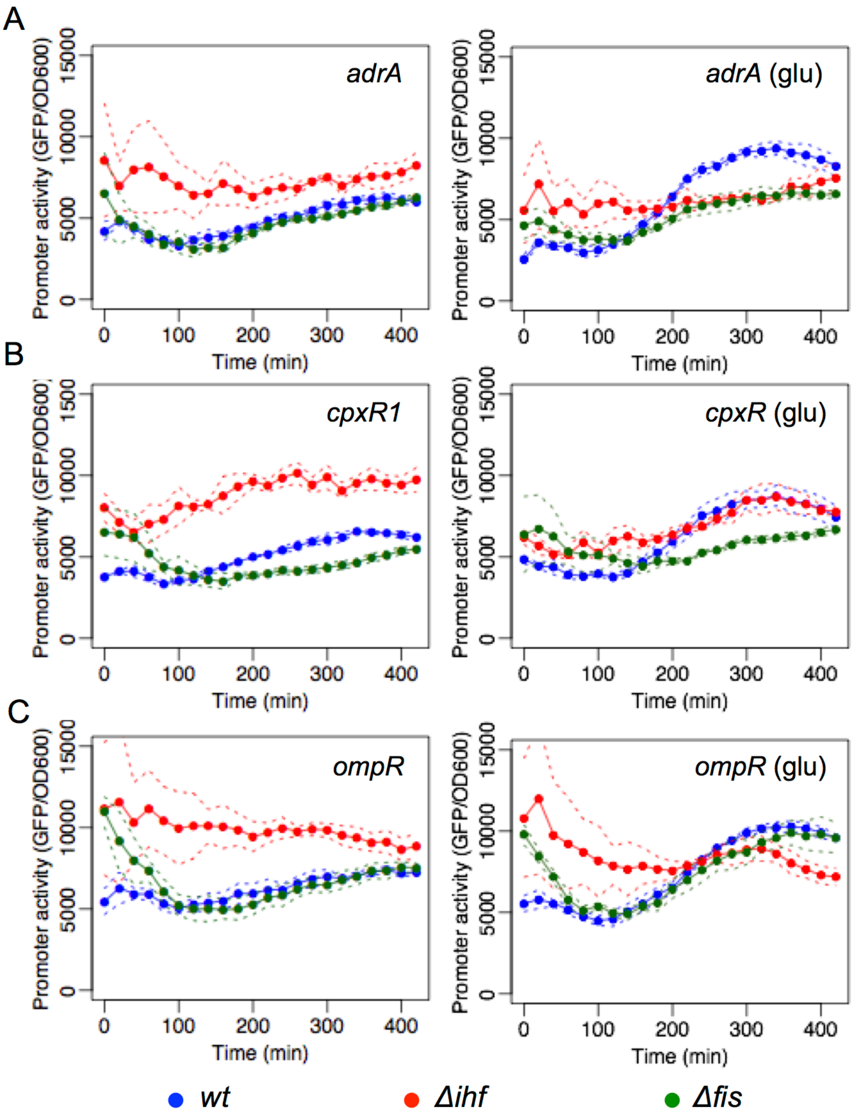

Figure S5. Effect of CRP, IHF and Fis GRs over the promoter activity of *adrA*, *cpxR* and *ompR*. Promoter activity assay of (A) pMR1-*PadrA,* (B) pMR1-*PcpxR,* and (C) pMR1-*PompR* were evaluated in *E. coli* BW25113 *wild-type* (blue line), Δ*ihf* (red line) and Δ*fis* (green line) in 96well plate as described in methods in the absence (left panel) or presence (right panel) of 0.4% of glucose. GFP fluorescence was measured every 20 minutes for 8 hours growth at 37 ºC in static conditions (normalized by OD600). Solid lines represent the mean of three independent experiments, while dashed lines are the upper and lower limits of SE.

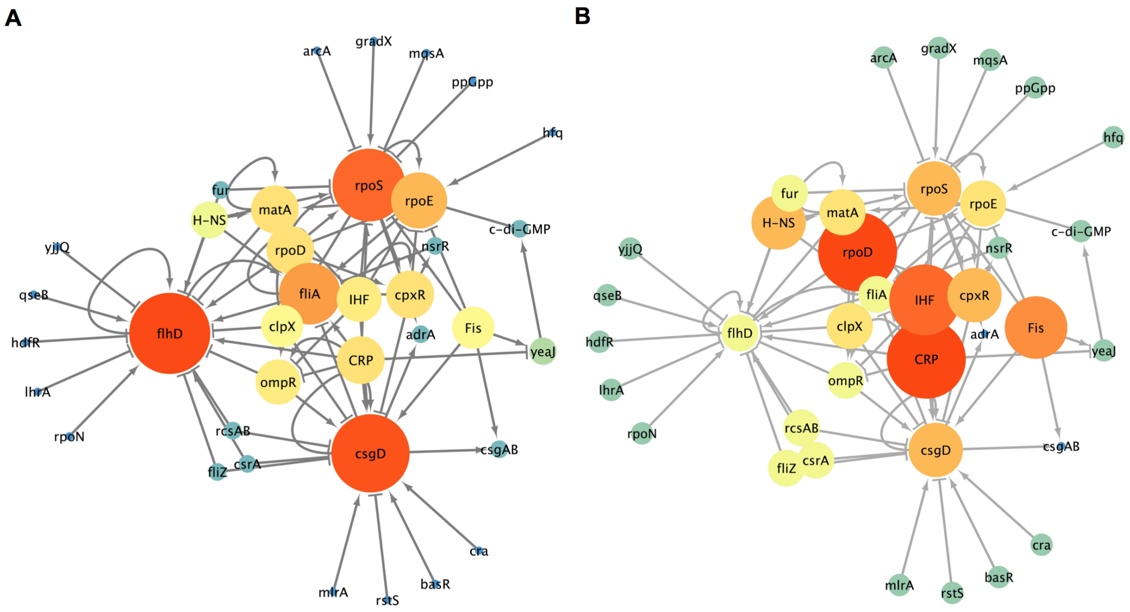

Figure S6. Flagella-biofilm transcriptional regulatory network with new interactions added. The principal nodes and paths, which drive the flagella-biofilm network upon the introduction of seven new interactions were analyzed using Cytoscape 3.4.1. The network was analyzed by using degree (A) and out-degree (B). Size of the nodes (circles) indicates number of interactions of the nodes. Color scale are from blue-green-yellow to orange indicating from low to high degree values.

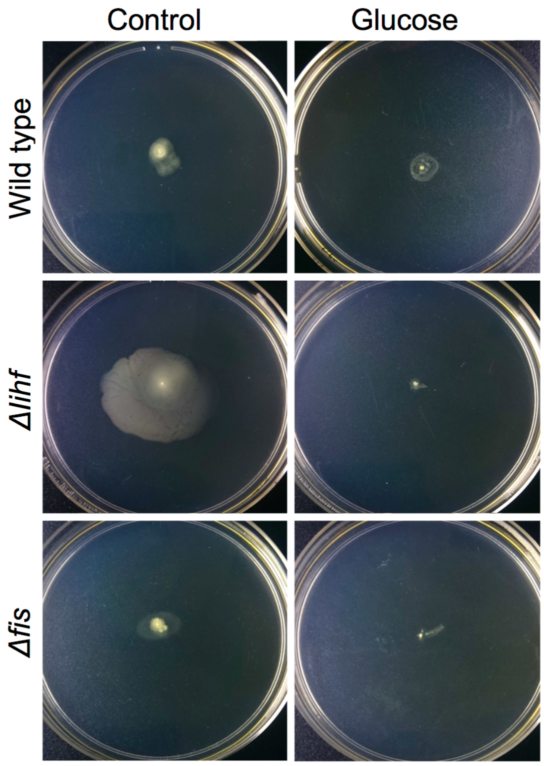

Figure S7. Effect of GRs in the motility program at 18h. Motility phenotype of *E. coli* BW25113 wild-type and mutant strains were evaluated by cell motility assay at 18h. in the presence or absence of glucose as depicted. Divergent motility capability is observed between the different conditions, proving the effect of the GRs CRP, IHF and Fis to modulate the motility program. The results are representative of 3 independent experiments.

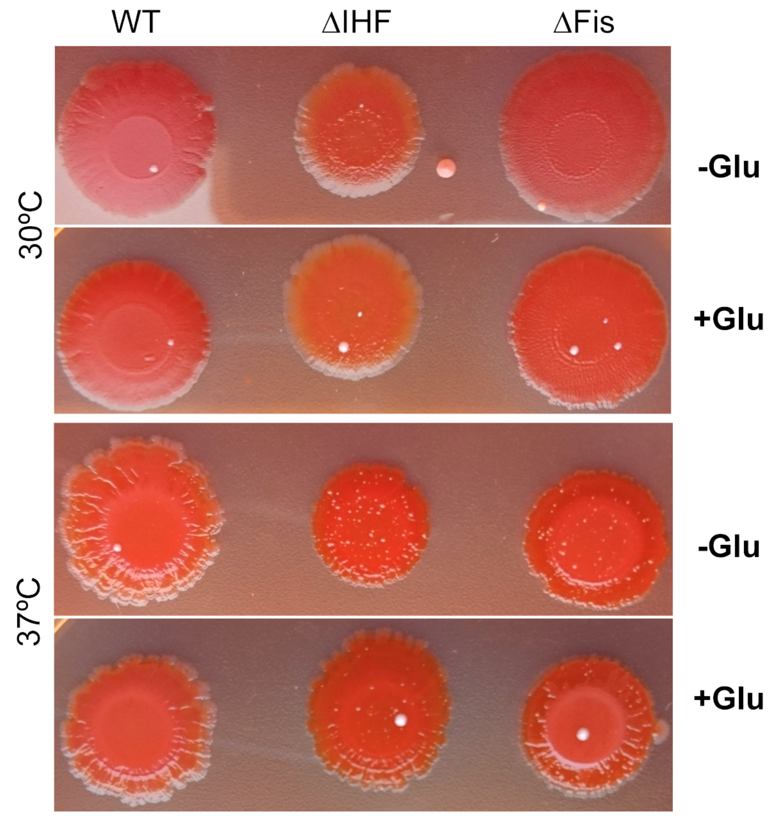

Figure S8. Capability of *E. coli* and mutant strains to develop mature biofilm. Mature biofilm formation of *E. coli* BW25113 *wt*, Δ*ihf* and Δ*fis* strains were perform using Congo red plate assay. Comparisons of the mature biofilm morphological characteristics of wild-type and mutant strains at 30 ºC and 37˚C in the presence or absence of glucose is shown. The results are representative of 3 independent experiments.

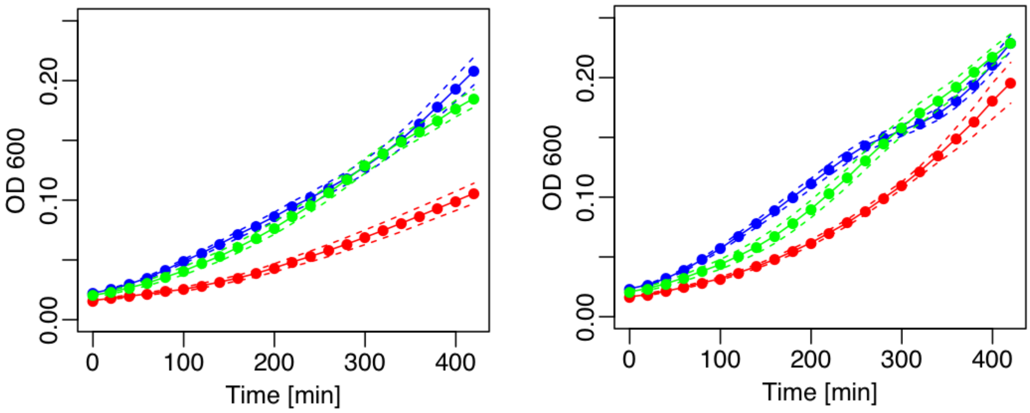

Figure S9. Growth curve of *E. coli* wild-type and mutant strains under the conditions used for promoter analysis. Experiments were performed in the absence (A) or presence (B) of 0.4% of glucose. Optical density at 600nm (OD600) was measured every 20 minutes over 8 hours growth at 37 ºC in static conditions. *E. coli* wild type (blue), ihf (red) and fis (green) mutants are analyzed. Solid lines represent the mean of three independent experiments while dashed lines are the upper and lower limits of SE.

Table S1. Regulatory interaction used for network analysis.

Effector	Effect	Terget	Bibliography
arcA	inhibits	rpoS	[2]
basR	activates	csgD	[3]
c-di-GMP	activates	rpoS	[4]
clpX	inhibits	csgD	[5]
clpX	inhibits	flhD	[6]
clpX	inhibits	rpoS	[7,8]
cpxR	activates	cpxR	[9-11]
cpxR	inhibits	csgD	[12-14]
cpxR	activates	nsrR	[12]
cpxR	inhibits	rpoE	[15]
cra	activates	csgD	[16]
CRP	activates	csgD	[17]
CRP	activates	flhD	[18]
CRP	inhibits	ompR	[19]
CRP	activates	ompR	[20]
CRP	inhibits	rpoS	[21]
CRP	activates	rpoS	[21]
csgD	activates	adrA	[13,22]
csgD	activates	csgAB	[13,23]
csgD	activates	csgD	[17]
csgD	inhibits	fliA	[12,24]
csrA	inhibits	csgD	[13]
csrA	activates	flhD	[25,26]
Fis	activates	csgAB	[27]
flhD	activates	flhD	[28]
flhD	activates	fliA	[28]
fliA	activates	flhD	[28]

fliZ	inhibits	csgD	29
fliZ	inhibits	flhD	29
fur	inhibits	flhD	30
fur	inhibits	rpoS	31,32
gradX	activates	rpoS	33
H-NS	activates	flhD	18
H-NS	activates	fliA	34
H-NS	activates	matA	35
H-NS	activates	rpoS	21
hdfR	inhibits	flhD	36
hfq	activates	rpoE	37
IHF	activates	csgD	13
IHF	activates	matA	35
IHF	inhibits	ompR	13
IHF	inhibits	rpoS	38
lhrA	inhibits	flhD	39
matA	inhibits	flhD	40
matA	inhibits	fliA	40
matA	activates	matA	40
mlrA	activates	csgD	13
mqsA	inhibits	rpoS	41
nsrR	inhibits	fliA	42
ompR	activates	csgD	14
ompR	inhibits	flhD	43,44
ppGpp	inhibits	rpoS	21,45
qseB	activates	flhD	46
rcsAB	inhibits	csgD	47
rcsAB	inhibits	flhD	43,48
rpoD	activates	cpxR	11,49
rpoD	activates	flhD	50

rpoD	activates	fliA	[49,50]
rpoD	activates	ompR	[51]
rpoD	activates	rpoE	[49,52]
rpoD	activates	rpoS	[49,53]
rpoE	activates	fliA	[54]
rpoE	activates	matA	[55,56]
rpoE	activates	rpoE	[57]
rpoN	activates	flhD	[58]
rpoS	activates	adrA	[59]
rpoS	activates	clpX	[60]
rpoS	activates	cpxR	[12]
rpoS	activates	rpoE	[57]
rstS	inhibits	csgD	[13]
yeaJ	activates	c-di-GMP	[61]
yjjQ	inhibits	flhD	[29,61]

References

1 Munch, R. *et al.* Virtual Footprint and PRODORIC: an integrative framework for regulon prediction in prokaryotes. *Bioinformatics* **21**, 4187-4189, doi:10.1093/bioinformatics/bti635 (2005).

2 Mika, F. & Hengge, R. A two-component phosphotransfer network involving ArcB, ArcA, and RssB coordinates synthesis and proteolysis of sigmaS (RpoS) in E. coli. *Genes & development* **19**, 2770-2781, doi:10.1101/gad.353705 (2005).

3 Ogasawara, H., Shinohara, S., Yamamoto, K. & Ishihama, A. Novel regulation targets of the metal-response BasS-BasR two-component system of Escherichia coli. *Microbiology* **158**, 1482-1492, doi:10.1099/mic.0.057745-0 (2012).

4 Weber, H., Pesavento, C., Possling, A., Tischendorf, G. & Hengge, R. Cyclic-di-GMP-mediated signalling within the sigma network of Escherichia coli. *Mol Microbiol* **62**, 1014-1034, doi:10.1111/j.1365-2958.2006.05440.x (2006).

5 Simm, R., Remminghorst, U., Ahmad, I., Zakikhany, K. & Römling, U. A role for the EAL-like protein STM1344 in regulation of CsgD expression and motility in Salmonella enterica serovar Typhimurium. *J Bacteriol* **191**, 3928-3937, doi:10.1128/jb.00290-09 (2009).

6 Kitagawa, R., Takaya, A. & Yamamoto, T. Dual regulatory pathways of flagellar gene expression by ClpXP protease in enterohaemorrhagic Escherichia coli. *Microbiology* **157**, 3094-3103, doi:10.1099/mic.0.051151-0 (2011).

7 Hengge-Aronis, R. Recent insights into the general stress response regulatory network in Escherichia coli. *Journal of molecular microbiology and biotechnology* **4**, 341-346 (2002).
8 Schweder, T., Lee, K. H., Lomovskaya, O. & Matin, A. Regulation of Escherichia coli starvation sigma factor (sigma s) by ClpXP protease. *J Bacteriol* **178**, 470-476 (1996).
9 Otto, K. & Silhavy, T. J. Surface sensing and adhesion of Escherichia coli controlled by the Cpx-signaling pathway. *Proc Natl Acad Sci U S A* **99**, 2287-2292, doi:10.1073/pnas.042521699 (2002).
10 Raivio, T. L. & Silhavy, T. J. The sigmaE and Cpx regulatory pathways: overlapping but distinct envelope stress responses. *Curr Opin Microbiol* **2**, 159-165, doi:10.1016/s1369-5274(99)80028-9 (1999).
11 De Wulf, P., Kwon, O. & Lin, E. C. The CpxRA signal transduction system of Escherichia coli: growth-related autoactivation and control of unanticipated target operons. *J Bacteriol* **181**, 6772-6778 (1999).
12 Dudin, O., Geiselmann, J., Ogasawara, H., Ishihama, A. & Lacour, S. Repression of flagellar genes in exponential phase by CsgD and CpxR, two crucial modulators of Escherichia coli biofilm formation. *J Bacteriol* **196**, 707-715, doi:10.1128/jb.00938-13 (2014).
13 Ogasawara, H., Yamada, K., Kori, A., Yamamoto, K. & Ishihama, A. Regulation of the Escherichia coli csgD promoter: interplay between five transcription factors. *Microbiology* **156**, 2470-2483, doi:10.1099/mic.0.039131-0 (2010).
14 Jubelin, G. *et al.* CpxR/OmpR interplay regulates curli gene expression in response to osmolarity in Escherichia coli. *J Bacteriol* **187**, 2038-2049, doi:10.1128/jb.187.6.2038-2049.2005 (2005).
15 De Wulf, P., McGuire, A. M., Liu, X. & Lin, E. C. Genome-wide profiling of promoter recognition by the two-component response regulator CpxR-P in Escherichia coli. *J Biol Chem* **277**, 26652-26661, doi:10.1074/jbc.M203487200 (2002).
16 Reshamwala, S. M. & Noronha, S. B. Biofilm formation in Escherichia coli cra mutants is impaired due to down-regulation of curli biosynthesis. *Arch Microbiol* **193**, 711-722, doi:10.1007/s00203-011-0708-7 (2011).
17 Hufnagel, D. A. *et al.* The Catabolite Repressor Protein-Cyclic AMP Complex Regulates csgD and Biofilm Formation in Uropathogenic Escherichia coli. *J Bacteriol* **198**, 3329-3334, doi:10.1128/jb.00652-16 (2016).
18 Soutourina, O. *et al.* Multiple control of flagellum biosynthesis in Escherichia coli: role of H-NS protein and the cyclic AMP-catabolite activator protein complex in transcription of the flhDC master operon. *J Bacteriol* **181**, 7500-7508 (1999).
19 Pratt, L. A., Hsing, W., Gibson, K. E. & Silhavy, T. J. From acids to osmZ: multiple factors influence synthesis of the OmpF and OmpC porins in Escherichia coli. *Mol Microbiol* **20**, 911-917 (1996).
20 Ebright, R. H. Transcription activation at Class I CAP-dependent promoters. *Mol Microbiol* **8**, 797-802 (1993).
21 Hengge-Aronis, R. Signal transduction and regulatory mechanisms involved in control of the sigma(S) (RpoS) subunit of RNA polymerase. *Microbiol Mol Biol Rev* **66**, 373-395, table of contents (2002).
22 Römling, U. Characterization of the rdar morphotype, a multicellular behaviour in Enterobacteriaceae. *Cell Mol Life Sci* **62**, 1234-1246, doi:10.1007/s00018-005-4557-x (2005).

23 Perni, S., Preedy, E. C., Landini, P. & Prokopovich, P. Influence of csgD and ompR on Nanomechanics, Adhesion Forces, and Curli Properties of E. coli. *Langmuir* **32**, 7965-7974, doi:10.1021/acs.langmuir.6b02342 (2016).
24 Ogasawara, H., Yamamoto, K. & Ishihama, A. Role of the biofilm master regulator CsgD in cross-regulation between biofilm formation and flagellar synthesis. *J Bacteriol* **193**, 2587-2597, doi:10.1128/jb.01468-10 (2011).
25 Yakhnin, H. *et al.* Complex regulation of the global regulatory gene csrA: CsrA-mediated translational repression, transcription from five promoters by $E\sigma^{70}$ and Eσ(S), and indirect transcriptional activation by CsrA. *Mol Microbiol* **81**, 689-704, doi:10.1111/j.1365-2958.2011.07723.x (2011).
26 Wei, B. L. *et al.* Positive regulation of motility and flhDC expression by the RNA-binding protein CsrA of Escherichia coli. *Mol Microbiol* **40**, 245-256 (2001).
27 Saldaña, Z. *et al.* Synergistic role of curli and cellulose in cell adherence and biofilm formation of attaching and effacing Escherichia coli and identification of Fis as a negative regulator of curli. *Environ Microbiol* **11**, 992-1006, doi:10.1111/j.1462-2920.2008.01824.x (2009).
28 Liu, X. & Matsumura, P. The FlhD/FlhC complex, a transcriptional activator of the Escherichia coli flagellar class II operons. *J Bacteriol* **176**, 7345-7351 (1994).
29 Pesavento, C. *et al.* Inverse regulatory coordination of motility and curli-mediated adhesion in Escherichia coli. *Genes & development* **22**, 2434-2446, doi:10.1101/gad.475808 (2008).
30 Kurabayashi, K., Agata, T., Asano, H., Tomita, H. & Hirakawa, H. Fur Represses Adhesion to, Invasion of, and Intracellular Bacterial Community Formation within Bladder Epithelial Cells and Motility in Uropathogenic Escherichia coli. *Infect Immun* **84**, 3220-3231, doi:10.1128/iai.00369-16 (2016).
31 Guillemet, M. L. & Moreau, P. L. Fur-dependent detoxification of organic acids by rpoS mutants during prolonged incubation under aerobic, phosphate starvation conditions. *J Bacteriol* **190**, 5567-5575, doi:10.1128/jb.00577-08 (2008).
32 Lelong, C., Rolland, M., Louwagie, M., Garin, J. & Geiselmann, J. Mutual regulation of Crl and Fur in Escherichia coli W3110. *Molecular & cellular proteomics : MCP* **6**, 660-668, doi:10.1074/mcp.M600192-MCP200 (2007).
33 Tucker, D. L. *et al.* Genes of the GadX-GadW regulon in Escherichia coli. *J Bacteriol* **185**, 3190-3201 (2003).
34 Kim, E. A. & Blair, D. F. Function of the Histone-Like Protein H-NS in Motility of Escherichia coli: Multiple Regulatory Roles Rather than Direct Action at the Flagellar Motor. *J Bacteriol* **197**, 3110-3120, doi:10.1128/jb.00309-15 (2015).
35 Martínez-Santos, V. I., Medrano-López, A., Saldaña, Z., Girón, J. A. & Puente, J. L. Transcriptional regulation of the ecp operon by EcpR, IHF, and H-NS in attaching and effacing Escherichia coli. *J Bacteriol* **194**, 5020-5033, doi:10.1128/jb.00915-12 (2012).
36 Ko, M. & Park, C. H-NS-Dependent regulation of flagellar synthesis is mediated by a LysR family protein. *J Bacteriol* **182**, 4670-4672 (2000).
37 Guisbert, E., Rhodius, V. A., Ahuja, N., Witkin, E. & Gross, C. A. Hfq modulates the sigmaE-mediated envelope stress response and the sigma32-mediated cytoplasmic stress response in Escherichia coli. *J Bacteriol* **189**, 1963-1973, doi:10.1128/jb.01243-06 (2007).
38 Mangan, M. W. *et al.* The integration host factor (IHF) integrates stationary-phase and virulence gene expression in Salmonella enterica serovar Typhimurium. *Mol Microbiol* **59**, 1831-1847, doi:10.1111/j.1365-2958.2006.05062.x (2006).

39 Habdas, B. J., Smart, J., Kaper, J. B. & Sperandio, V. The LysR-type transcriptional regulator QseD alters type three secretion in enterohemorrhagic Escherichia coli and motility in K-12 Escherichia coli. *J Bacteriol* **192**, 3699-3712, doi:10.1128/jb.00382-10 (2010).
40 Lehti, T. A., Bauchart, P., Dobrindt, U., Korhonen, T. K. & Westerlund-Wikström, B. The fimbriae activator MatA switches off motility in Escherichia coli by repression of the flagellar master operon flhDC. *Microbiology* **158**, 1444-1455, doi:10.1099/mic.0.056499-0 (2012).
41 Wang, X. *et al.* Antitoxin MqsA helps mediate the bacterial general stress response. *Nat Chem Biol* **7**, 359-366, doi:10.1038/nchembio.560 (2011).
42 Partridge, J. D., Bodenmiller, D. M., Humphrys, M. S. & Spiro, S. NsrR targets in the Escherichia coli genome: new insights into DNA sequence requirements for binding and a role for NsrR in the regulation of motility. *Mol Microbiol* **73**, 680-694, doi:10.1111/j.1365-2958.2009.06799.x (2009).
43 Samanta, P., Clark, E. R., Knutson, K., Horne, S. M. & Prüß, B. M. OmpR and RcsB abolish temporal and spatial changes in expression of flhD in Escherichia coli biofilm. *BMC Microbiol* **13**, 182, doi:10.1186/1471-2180-13-182 (2013).
44 Shin, S. & Park, C. Modulation of flagellar expression in Escherichia coli by acetyl phosphate and the osmoregulator OmpR. *J Bacteriol* **177**, 4696-4702 (1995).
45 Lange, R., Fischer, D. & Hengge-Aronis, R. Identification of transcriptional start sites and the role of ppGpp in the expression of rpoS, the structural gene for the sigma S subunit of RNA polymerase in Escherichia coli. *J Bacteriol* **177**, 4676-4680 (1995).
46 Egler, M., Grosse, C., Grass, G. & Nies, D. H. Role of the extracytoplasmic function protein family sigma factor RpoE in metal resistance of Escherichia coli. *J Bacteriol* **187**, 2297-2307, doi:10.1128/jb.187.7.2297-2307.2005 (2005).
47 Shimada, T. *et al.* A novel regulator RcdA of the csgD gene encoding the master regulator of biofilm formation in Escherichia coli. *Microbiologyopen* **1**, 381-394, doi:10.1002/mbo3.42 (2012).
48 Lehti, T. A., Heikkinen, J., Korhonen, T. K. & Westerlund-Wikström, B. The response regulator RcsB activates expression of Mat fimbriae in meningitic Escherichia coli. *J Bacteriol* **194**, 3475-3485, doi:10.1128/jb.06596-11 (2012).
49 Shimada, T., Yamazaki, Y., Tanaka, K. & Ishihama, A. The whole set of constitutive promoters recognized by RNA polymerase RpoD holoenzyme of Escherichia coli. *PLoS One* **9**, e90447, doi:10.1371/journal.pone.0090447 (2014).
50 Isalan, M. *et al.* Evolvability and hierarchy in rewired bacterial gene networks. *Nature* **452**, 840-845, doi:10.1038/nature06847 (2008).
51 Yamamoto, K. *et al.* Functional characterization in vitro of all two-component signal transduction systems from Escherichia coli. *J Biol Chem* **280**, 1448-1456, doi:10.1074/jbc.M410104200 (2005).
52 Azam, T. A. & Ishihama, A. Twelve species of the nucleoid-associated protein from Escherichia coli. Sequence recognition specificity and DNA binding affinity. *J Biol Chem* **274**, 33105-33113 (1999).
53 Klauck, E., Typas, A. & Hengge, R. The sigmaS subunit of RNA polymerase as a signal integrator and network master regulator in the general stress response in Escherichia coli. *Science progress* **90**, 103-127 (2007).
54 Bury-Moné, S. *et al.* Global analysis of extracytoplasmic stress signaling in Escherichia coli. *PLoS Genet* **5**, e1000651, doi:10.1371/journal.pgen.1000651 (2009).
55 Beloin, C. *et al.* Global impact of mature biofilm lifestyle on Escherichia coli K-12 gene expression. *Mol Microbiol* **51**, 659-674 (2004).

56 Dartigalongue, C., Missiakas, D. & Raina, S. Characterization of the Escherichia coli sigma E regulon. *J Biol Chem* **276**, 20866-20875, doi:10.1074/jbc.M100464200 (2001).
57 Peano, C. *et al.* Characterization of the Escherichia coli σ(S) core regulon by Chromatin Immunoprecipitation-sequencing (ChIP-seq) analysis. *Scientific reports* **5**, 10469, doi:10.1038/srep10469 (2015).
58 Dong, T., Yu, R. & Schellhorn, H. Antagonistic regulation of motility and transcriptome expression by RpoN and RpoS in Escherichia coli. *Mol Microbiol* **79**, 375-386, doi:10.1111/j.1365-2958.2010.07449.x (2011).
59 White-Ziegler, C. A. *et al.* Low temperature (23 degrees C) increases expression of biofilm-, cold-shock- and RpoS-dependent genes in Escherichia coli K-12. *Microbiology* **154**, 148-166, doi:10.1099/mic.0.2007/012021-0 (2008).
60 Repoila, F., Majdalani, N. & Gottesman, S. Small non-coding RNAs, co-ordinators of adaptation processes in Escherichia coli: the RpoS paradigm. *Mol Microbiol* **48**, 855-861 (2003).
61 Sommerfeldt, N. *et al.* Gene expression patterns and differential input into curli fimbriae regulation of all GGDEF/EAL domain proteins in Escherichia coli. *Microbiology* **155**, 1318-1331, doi:10.1099/mic.0.024257-0 (2009).
